# Mitochondrial polymorphism m.3017C>T of SHLP6 relates to heterothermy

**DOI:** 10.3389/fphys.2023.1207620

**Published:** 2023-08-21

**Authors:** Sarah V. Emser, Clemens P. Spielvogel, Eva Millesi, Ralf Steinborn

**Affiliations:** ^1^ Department of Behavioral and Cognitive Biology, University of Vienna, Vienna, Austria; ^2^ Genomics Core Facility, VetCore, University of Veterinary Medicine, Vienna, Austria; ^3^ Department of Biomedical Imaging and Image-Guided Therapy, Division of Nuclear Medicine, Medical University of Vienna, Vienna, Austria; ^4^ Department of Microbiology, Immunobiology and Genetics, University of Vienna, Vienna, Austria

**Keywords:** daily torpor, hibernation, mitogenomics, mitochondrial-derived peptide (MDP), micropeptide, SHLP6, rodents, extended vertebrate mitochondrial genetic code

## Abstract

Heterothermic thermoregulation requires intricate regulation of metabolic rate and activation of pro-survival factors. Eliciting these responses and coordinating the necessary energy shifts likely involves retrograde signalling by mitochondrial-derived peptides (MDPs). Members of the group were suggested before to play a role in heterothermic physiology, a key component of hibernation and daily torpor. Here we studied the mitochondrial single-nucleotide polymorphism (SNP) m.3017C>T that resides in the evolutionarily conserved gene *MT-SHLP6.* The substitution occurring in several mammalian orders causes truncation of SHLP6 peptide size from twenty to nine amino acids. Public mass spectrometric (MS) data of human SHLP6 indicated a canonical size of 20 amino acids, but not the use of alternative translation initiation codons that would expand the peptide. The shorter isoform of SHLP6 was found in heterothermic rodents at higher frequency compared to homeothermic rodents (*p* < 0.001). In heterothermic mammals it was associated with lower minimal body temperature (*T*_*b*_, *p* < 0.001). In the thirteen-lined ground squirrel, brown adipose tissue—a key organ required for hibernation, showed dynamic changes of the steady-state transcript level of *mt-Shlp6*. The level was significantly higher before hibernation and during interbout arousal and lower during torpor and after hibernation. Our finding argues to further explore the mode of action of SHLP6 size isoforms with respect to mammalian thermoregulation and possibly mitochondrial retrograde signalling.

## 1 Introduction

Mammalian species use heterothermy as a very effective energy-saving strategy to overcome harsh environmental conditions (Morales et al., 2021). In addition to maintaining a *T*_*b*_ of around 37°C during activity, heterothermic species are capable to enter controlled, hypometabolic phases of less or more than 24 h termed daily torpor or hibernation (multiday torpor), respectively (Barnes et al., 1986; Hume et al., 2002; Sheriff et al., 2013; Ruf and Geiser, 2015). Heterothermy often refers to a regular adaptation to seasonality over several months but can also be used as a response to unpredictable environmental conditions and emergency situations (Nowack et al., 2020). Despite stratification into the phenotypes of daily and multiday torpor, patterns of heterothermic responses are highly diverse (Ruf and Geiser, 2015; Levesque et al., 2016; Nespolo et al., 2021). Currently, heterothermy is considered a preceding state of endothermy in early mammals, with mainly passive temperature regulation and short or longer-term metabolic upregulation. This view regards daily torpor and hibernation as derived states of early heterothermy (Nowack et al., 2020).

Torpor can be viewed as series of physiological adaptations including reduction of *T*_*b*_ and metabolic rate (*MR*) (Ruf and Geiser, 2015), electro-cerebral inactivity (Andrews, 2019) and others. While foraging is continued in species that employ daily torpor, hibernation requires preparation such as accumulation of body fat or food stores (Humphries et al., 2003; Siutz et al., 2016), modification of reproductive and digestive systems and reliably favourable conditions for quick recovery (Barnes et al., 1986; Hume et al., 2002; Sheriff et al., 2013). Hibernation is interspersed by short obligate euthermic phases termed interbout arousals whose duration is inversely related to torpid *MR* (Ruf et al., 2022). The phases are triggered by accumulation or depletion of metabolites to eliminate cellular damage (Galster and Morrison, 1975; van Breukelen and Martin, 2001; Wiersma et al., 2018). Although necessary, interbout arousal is energetically expensive (Carey et al., 2003; Karpovich et al., 2009) and associated with increased cellular damage and production of reactive oxygen species (Carey et al., 2000; Brown and Staples, 2011; Nowack et al., 2019).

The active metabolic suppression during torpor coincides with a reversible repression of mitochondrial respiration down to 30% (Staples and Brown, 2008; Brown et al., 2012; Mathers et al., 2017). Mitochondria are responsible for oxidative phosphorylation (OXPHOS) and ATP production. Nucleoids of this cellular organelle have a uniform size and frequently contain a single copy of a compact circular genome, for example, on average 1.4 copies of a 16.5 kb circular genome (mitogenome) in case of man (Kukat et al., 2011). The common mitogenome in Vertebrata encodes 13 essential protein subunits of the respiratory chain, two rRNAs, 22 tRNAs (Ren et al., 2023) and several small open-reading frame (sORF)-derived micropeptides, commonly defined as polypeptides with a relatively arbitrary length of less than 100 to 150 amino acids [references in (Sousa and Farkas, 2018)], that represent the low end of the canonical protein spectrum (Schlesinger and Elsasser, 2022). Currently, the latter subgroup of micropeptides termed MDPs, consists of Humanin (Hashimoto et al., 2001), Gau [gene antisense ubiquitous (Faure et al., 2011)], MOTS-c [mitochondrial ORF of the 12S rRNA type-c (Lee et al., 2015)], SHLP1-6 [small Humanin-like peptides 1 to 6 (Cobb et al., 2016)], SHMOOSE [Small Human Mitochondrial ORF Over SErine tRNA (Miller et al., 2022)] and MTALTND4 [mitochondrial alternative ND4 protein (Kienzle et al., 2023)]. At least for a species which does not have a nuclear insertion of mitochondrial origin [NUMT (Wei et al., 2022)], mtDNA is the exclusive source for MDP coding, as, for example, in the cases of rat Humanin and rat MOTS-c (Paharkova et al., 2015). Cytoplasmic tRNA import (Rubio et al., 2008; Jeandard et al., 2019; Kienzle et al., 2023) and the use of alternative start codons (compiled by NCBI at https://www.ncbi.nlm.nih.gov/Taxonomy/Utils/wprintgc.cgi) expand the coding capacity of the mitochondrial genetic code, with consequences for MDP size (see below, Figures 3, 4F) and also for fine-tuning of complex biological systems that would be highly likely given the uniquely suited small size of MDPs.

Physiological extremes of hypothermic torpor and rapid arousal requires tremendous resilience of the cardiovascular and nervous systems to counteract the reduction and resumption of blood flow (Dave et al., 2012; Andrews, 2019). Contribution to the heterothermic phenotype can involve alteration of transcriptional and/or posttranscriptional regulation, post-translational modification (PTM) that is subject of dynamic regulation during hibernation (Tessier et al., 2017; Mathers and Staples, 2019), and microproteins such as Humanin, MOTS-c and SHLP2 (Supplementary Table S1). MOTS-c was the first MDP identified as a regulator of thermal homeostasis (Lu et al., 2019). Administration of MOTS-c enhanced cold tolerance by boosting two mechanism that are needed for heterothermy, namely white fat browning and thermogenic gene expression in brown adipose tissue, a well-known organ for thermogenesis (Lu et al., 2019). It downregulates circulating metabolites that are associated with type-2 diabetes and obesity, enhances glucose tolerance and insulin sensitivity (Lee et al., 2015; Kim et al., 2018; Benayoun and Lee, 2019) and functions as a host-defence peptide according to a preprint (Rice, 2023). Mitochondrial retrograde signalling, a major form of mitochondria to nucleus crosstalk that is activated by altered mitochondrial function under normal or pathophysiological conditions and enables reprogramming of nuclear gene expression (Liu and Butow, 2006; Granat et al., 2020), is the likely route of MOTS-c-mediated metaboloprotection. Humanin, the MDP archetype, shows elevated transcript and peptide expression in the brain cortex during hibernation pointing to protection of delicate brain tissues and neuronal connections against hibernation-associated stresses (Szereszewski and Storey, 2019).

Involvement in modulating endothermic thermoregulation may also be assumed for SHLP6, another member of the MDP group that is inducible by high-intensity exercise (Woodhead et al., 2020); Supplementary Table S1). Exercise generates high levels of acute mechanical, metabolic and thermoregulatory stress (Holloszy and Coyle, 1984; Wagner, 1991; Hamilton and Booth, 2000) and causes an adaptive response that might explain the inverse relationship with conditions such as metabolic and cardiovascular disease (Wenger and Bell, 1986; Harber et al., 2017; Laukkanen and Kujala, 2018). A similar inverse relationship is encountered during hibernation when the organism adjusts to a drastic change in physiology (Kurtz et al., 2021). SHLP6 is one of the most widely distributed MDPs with predictions ranging from mammals (Emser et al., 2021) to other vertebrates and some spiders (Supplementary Table S2). Across tissues of the mouse it shows pronounced differences in expression ranging from high abundance in liver and kidney, over weak expression in heart, brain, spleen and prostate, down to a lack in testes and muscle (Cobb et al., 2016). Being an apoptosis enhancer (Cobb et al., 2016), SHLP6 could be involved in organ shrinkage, hence facilitate reduction of energy consumption during torpor or thermoregulation (Hume et al., 2002).

Other members of the SHLP family are antagonistic to the apoptosis inducer SHLP6. They promote cell proliferation [SHLP4 (Cobb et al., 2016)], improve cell viability, reduce cell apoptosis, and share protective effects with Humanin [SHLP2 and SHLP3 (Cobb et al., 2016)]. SHLP2, in addition, promotes thermogenesis in interscapular brown adipose tissue (Kim et al., 2023). SHMOOSE boosts mitochondrial oxygen consumption, modifies energy signalling and metabolism in the central nervous system as well as reduces mitochondrial superoxide production (Miller et al., 2022). At least some of these functionalities would be essential for a torpid state that is accompanied by lower oxygen unloading to tissues. For example, attenuation of oxygen consumption mediated by MTALTND4 might be beneficial in this respect.

This study speculated on the role of SHLP6 in endothermic thermoregulation of rodents and added another MDP with a role in daily torpor and hibernation.

## 2 Materials and methods

### 2.1 Extraction and validation of mtDNA sequences from DNA- or RNA-seq data

DNA-, RNA- or exome sequencing data contained in the Sequence Read Archive (SRA) of NCBI were screened for the species with information on the use of endothermic heterothermy, but no information on the SHLP6-coding sequence. Sequence reads were downloaded to the Galaxy server [(Galaxy, 2022); https://usegalaxy.eu/], mapped to the closest mtDNA homologue available through BBmap (Bushnell, 2014) and aligned using rnaSPAdes [(Bushmanova et al., 2019); http://cab.spbu.ru/software/rnaspades/], respectively. For validation, we followed the proposed gold standard for mitogenome publishing and authentication (Sangster and Luksenburg, 2021). Phylogeny was reconstructed based on the mitochondrial mRNA-, rRNA- and tRNA-coding genes without partitioning using the maximum-likelihood approach performed in IQ-TREE [release 1.6.12 of 15 August 2019; (Nguyen et al., 2015; Kalyaanamoorthy et al., 2017; Hoang et al., 2018); http://iqtree.Cibiv.univie.ac.at/]. The sequences of mitogenomes were automatically annotated using the MITOchondrial genome annotation Server (MITOS) server [(Bernt et al., 2013); http://mitos.bioinf.uni-leipzig.de/index.py].

### 2.2 Amino acid sequences and phylogenetic reconstruction

Rodent orthologues of SHLP6 were identified using the sORF of human SHLP6 as query (GenBank accession number KX067784.1) for a Basic Local Alignment Search Tool (BLAST) search (target number: ≤5,000). Results were viewed in the Multiple Sequence Alignment Viewer of NCBI (https://www.ncbi.nlm.nih.gov/projects/msaviewer/) and translated using Seaview version 5.0.4 [(Gouy et al., 2010); https://doua.prabi.fr/software/seaview].

To illustrate length distribution of the SHLP6-encoding sORF, rodent phylogeny was reconstructed with Timetree version 5 [(Kumar et al., 2022); www.timetree.org] and edited in iTOL version 6 [(Letunic and Bork, 2021); https://itol.embl.de/].

### 2.3 Detection of SHLP6 peptide fragments in public MS data

To screen publicly available MS data for fragments of SHLP6, the commonly accepted amino acid sequences of human, rat and mouse SHLP6 were used as query. We also considered the option of an earlier translational start facilitated by the use of alternative codons for translation initiation used by mitochondria of Vertebrata (Sammet et al., 2010). In detail, evidence for human SHLP6 was searched in MS datasets integrated in the web-based targeted peptide search engine PepQuery 2 that was hosted by the Galaxy server [(Galaxy, 2022); https://usegalaxy.eu/]. PepQuery2 identifies or validates known and novel peptides of interest in any local or publicly available MS-based proteomics datasets [(Wen and Zhang, 2023); http://pepquery2.pepquery.org/]. The integrated datasets contained four PTM types, acetylation, glycosylation, phosphorylation and ubiquitinylation. Six MS datasets of mouse and rat were queried (MSV000083647, MSV000086732, MSV000088206, MSV000089856, MSV000091015, and MSV000091978).

### 2.4 Analysis of temporal transcript abundance patterns across hibernation states

Several tissues and activity states of the thirteen-lined ground squirrel served as model to analyse hibernation-associated transcript alteration using public RNA-seq data. In detail, RNA-seq reads of the SRA bioprojects PRJNA854159 (adrenal gland), PRJNA418486 (brain), PRJNA226612 (brown adipose tissue) and PRJNA702062 (liver) were downloaded to the Galaxy server. Data sets contained single-end sequence reads (brown adipose tissue: ∼100 bp) or paired-end reads (adrenal gland: ∼135 bp, brain and liver: ∼250 to 280 bp). Transcript abundance of the target genes *mt-Rnr2* (mitochondrially encoded 12S and 16S rRNA genes), sHumanin (Szereszewski and Storey, 2019) abbreviated as *mt-sHn*, and *mt-Shlp6* was quantified using Kallisto quant version 0.46.2 (Bray et al., 2016)*.* To obtain a sufficiently long target RNA that would be compatible with the read length of the RNA-seq library available for brown adipose tissue, we considered the expanded mitochondrial coding potential as outlined in the Introduction. In case of *mt-sHn*, we assumed misreading of the canonical stop codon that would increase the size of the peptide from 21 to 38 amino acids, thus expand the sORF to 114 nucleotides (nt). Details on sORF extension of *mt-Shlp6* (170 nt) are outlined in Section 3.3.

Variance-mean dependence of count data and their differential abundance based on negative binomial distribution were estimated by the DESeq2 software (Love et al., 2014).

### 2.5 Secondary structure prediction of human 16S rRNA

Secondary structure of the human 16S rRNA sequence (accession number NC_012920.1) was predicted without considering modulation by multiple proteins, in particular RNA-binding proteins (Georgakopoulos-Soares et al., 2022), using The Vienna RNA package RNAfold [(Gruber et al., 2008; Lorenz et al., 2011); http://rna.tbi.univie.ac.at/cgi-bin/RNAWebSuite/RNAfold.cgi] and UNAfold [(Markham and Zuker, 2008); http://www.unafold.org/].

### 2.6 Hydrophobicity and PTM prediction of SHLP6

The degree of hydrophobicity/hydrophilicity of human, mouse and rat SHLP6 was visualised as hydropathicity plot in Kyte-Doolittle scale [(Kyte and Doolittle, 1982); https://web.expasy.org/protscale/]. PTM was predicted using web servers with lowest threshold settings (phosphorylation: [(Wang et al., 2020); http://gps.biocuckoo.cn/online.php], methylation: [(Devall et al., 2016); https://methylsight.cu-bic.ca], glycosylation: [(Gupta and Brunak, 2002); https://services.healthtech.dtu.dk/services/NetNGlyc-1.0/, https://services.healthtech.dtu.dk/services/NetOGlyc-4.0/]: and small ubiquitin-like modifier (SUMO) addition (SUMOylation), and SUMO-interacting motif [(Beauclair et al., 2015); http://www.jassa.fr/index.php?m=jassa].

### 2.7 Peptide structure assessment and visualisation

Three-dimensional peptide structure was predicted using the deep-learning approach of ColabFold (Mirdita et al., 2022) that is based on AlphaFold2 (Jumper et al., 2021; Yang et al., 2023). Confidence of prediction was scored and ranked according to the predicted Local Distance Difference Test (*pLDDT*) value (Mariani et al., 2013) that was obtained by computing five models with three recycles for each amino acid sequence. To ensure reproducibility, structure prediction was performed with the default parameters of ColabFold. The top-scoring model was visualised using the structural analysis software iCn3D (Wang et al., 2022). Structures were aligned using the “residue by residue” option.

Alternative prediction of human SHLP6 folding was derived from AlphaFold Protein Structure Database of UniProt [(UniProt, 2023); https://alphafold.ebi.ac.uk].

### 2.8 Statistical analysis

Distribution of the m.3017C>T polymorphism, SHLP6 sizes and structure predictions across heterothermic and homeothermic rodents were compared using the chi-square test run in RStudio version 4.1.2 (RStudio Team, 2021).

Significance of the relationship between SHLP6 length and *T*_*b*_ or *MR* of the heterothermic mammalian species was evaluated using the Wilcoxon rank-sum test, a nonparametric test for two independent groups. The latter statistical analyses were performed in GraphPad Prism (version 9.5.1; GraphPad Software, Boston, MA, United States).

## 3 Results

### 3.1 Extraction and validation of mtDNA sequences from DNA- and RNA-seq data

Public sequencing data were used to extract mtDNA sequences of six heterothermic rodent species, namely *Acomys russatus*, *Otospermophilus beecheyi*, *Otospermophilus variegatus*, *Sicista betulina*, *Zapus hudsonius* and *Zapus princeps* (Table 1). Erroneous sequence assignation, *A. russatus* instead of *A. cahirinus*, was determined for the sequence reads SRR17216041 of the Bioproject PRJNA788430 (details not shown). The extracted mitogenomes were validated by phylogenetic reconstruction (Supplementary Figure S1), annotated and submitted to NCBI’s GenBank (Table 1).

**TABLE 1:** Novel mitogenomes extracted from publicly available sequence data.

Rodent species	SRA accession number	Sequencing technique	Mitogenome size (bp)	Accession ID (this study)
*Acomys russatus*	ERR4183373	WGS	16,218	BK063162
*Otospermophilus beecheyi*	SRR4180864	Exon capture	16,472	BK063161
*Otospermophilus variegatus*	SRR4180883	Exon capture	16,472	BK063160
*Sicista betulina* ^a^	SRR12432355	WGS	16,412	BK063159
*Zapus hudsonius*	SRR11434656	WGS	16,510	BK063158
*Zapus princeps*	SRR12430165	RNA-seq	15,740^b^	BK063163

^a^ Other mitogenome variants of this species were uploaded to the GenBank in parallel to this study.

^b^ Incomplete assembly (95%) due to repeated D-loop segments.

Sequence records provided by this study are accessible at the Third-Party Annotation (TPA) section of DDBJ/ENA/GenBank databases (https://www.ncbi.nlm.nih.gov/genbank/TPA.html).

### 3.2 Premature translation termination caused by m.3017C>T and relationship with parameters of heterothermy

**FIGURE 1:**
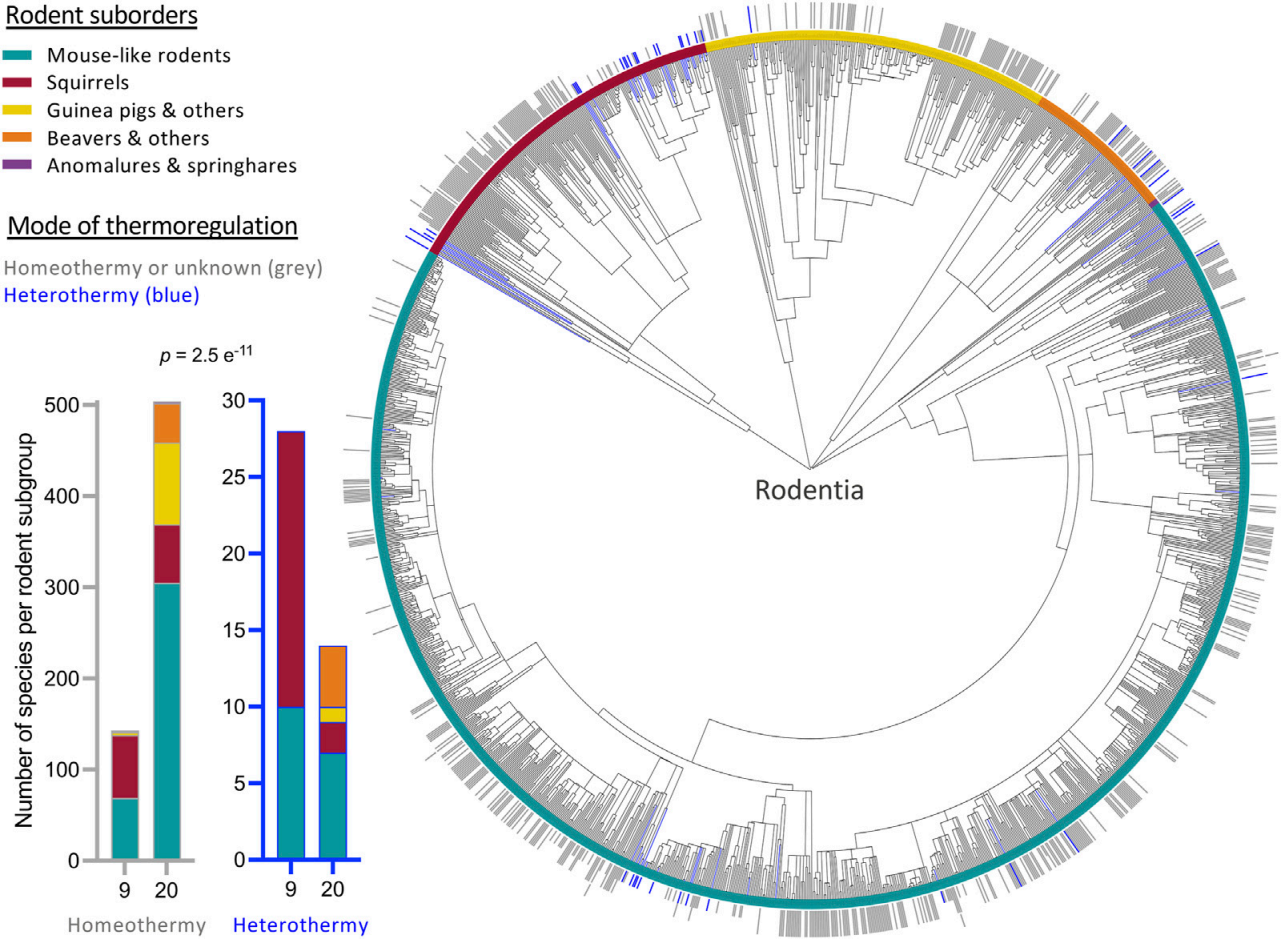
Phylogenetic tree depicting the distribution of SHLP6 peptide length across rodent species. Occurrence of heterothermy is highlighted in blue. 20 and 9mers of SHLP6 are depicted by a full bar outside of the tree or a half stroke, respectively. An additional short stroke highlights a species harbouring both length variants. In case no sequences were available at NCBI, the bar is left blank. Further research on little explored rodents is requested to increase the number of species with confidently classified as heterothermic (Ruf and Geiser, 2015). Statistic: chi-square test.

SHLP6 coding sequences of monotremes, specifically rodents, were found to be very conserved (Emser et al., 2021; Figures 6, 7). In relation to the mouse, homology at DNA and amino-acid levels ranged from 82% to 100% and 85%–100%, respectively (Supplementary Tables S3, S4). The nucleotide substitution that occurred in 22% of the rodent species currently sequenced, reduced the size of SHLP6 from twenty to nine amino acids. Analysis of sequences available for 34% species of the order Rodentia (686 of ∼2,000) including 42 species with proven heterothermy status (Ruf and Geiser, 2015) yielded a significantly higher frequency of the shorter peptide across heterotherms (Chi-square test: *p* < 0.001, Figure 1, Supplementary Tables S3, S4). Two suborders of the taxon Rodentia mirrored this significant relationship between SHLP6 length and endothermic thermoregulation (Myomorpha (mouse-like rodents): *p* = 0.0001 and Sciuromorpha (squirrels): *p* = 0.003). The remaining three taxonomic suborders, Castorimorpha, Anomaluromorpha and Hystricomorpha, lacked a critical number of heterothermic species for statistical analysis (*n* = 0 to 4, Figure 1). In contrast, the two length variants of SHLP6 were not found to be related to rodent’s or mammal’s cold adaption (*p* > 0.1; Supplementary Table S5) nor to heterothermic or homeothermic thermoregulation across other orders of the taxon Mammalia despite frequent occurrence (*p* > 0.1, Supplementary Table S6).

**FIGURE 2:**
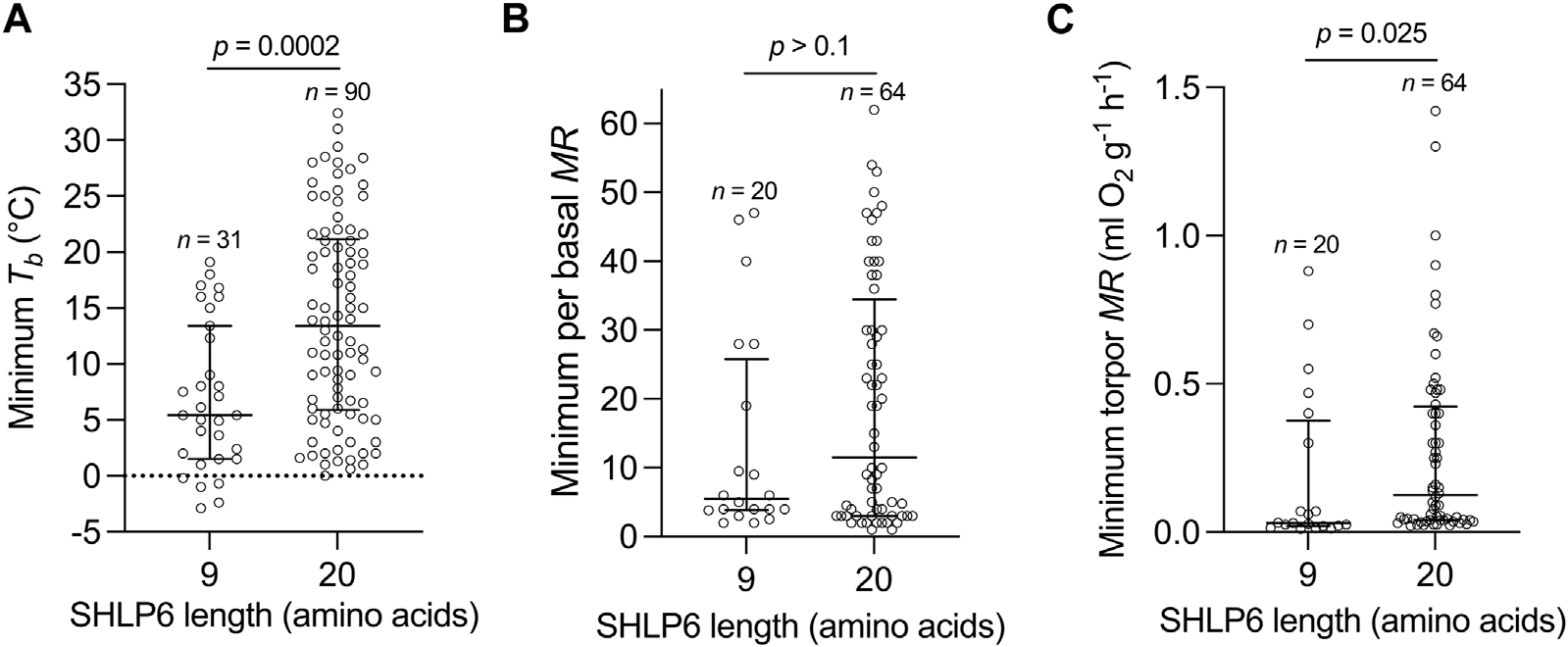
Length isoforms of SHLP6 in relation to physiological parameters of hibernation. Distribution of SHLP6 sizes across heterothermic mammals in relation to minimum *T*_*b*_ **(A)**, minimum torpid *MR* **(B)** and ratio of minimal to basal *MR* with *T*_*b*_ and *MR* values taken from Ruf and Geiser (2015) **(C)**. Analysis of significance: Wilcoxon rank-sum test, *T*_*b*_: body temperature, *MR*: metabolic rate.

Next, the physiological parameters minimal *T*_*b*_, minimum torpor *MR* and the ratio of minimal to basal *MR* of heterothermic mammals (Ruf and Geiser, 2015) were evaluated in relation to their SHLP6 size isoforms. Significances were found between the shorter SHLP6 isoform and minimal *T*_*b*_ (*p* = 0.0002) or minimum torpid *MR* (*p* = 0.025, Figure 2).

**FIGURE 3:**
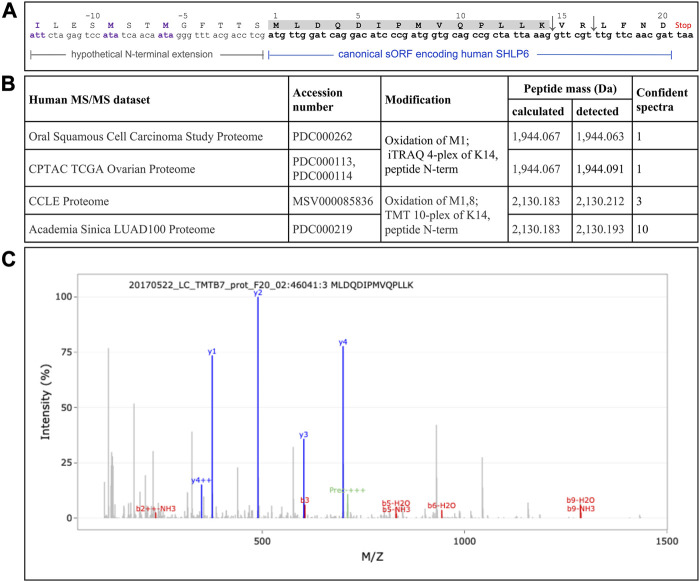
Sporadic detection of the non-enriched human SHLP6 using MS analysis **(A)** Hypothetical extension of the canonical human 20mer of SHLP6 predicted by Kienzle et al. (2023). This putative N-terminal extension is depicted by smaller font type. It was hypothesized to result from the use of the alternative translation initiation codons ATT and ATA depicted in violet. Positive or negative numbering refers to the canonical peptide or the hypothetical extension, respectively. The sequence of the cleaved 14mer peptide fragment, “MLDQDIPMVQPLLK”, is shaded. The two trypsin cleavage sites contained in the canonical sequence of human SHLP6, but not in the hypothetical N-terminal extension, are shown by the arrows. Origin of coding sequence: NCBI’s accession number J01415.2. **(B)** Details on the four human data sets that contained at least a single SHLP6-specific fragment. Notably, the same confident fragment was detected in all cases. **(C)** Example of a MS spectrum depicting the confident fragment “MLDQDIPMVQPLLK”. Blue and red signals represent “y” and “b” ions, respectively. Pre: precursor. The exemplary spectrogram was taken from the last of the data sets listed above.

Recently, the use of translation from alternative translation initiation codons applied by the mitochondrion got a renaissance, for example, illustrated by the mitochondrially encoded human peptides MTALTND4 and SHLP6 (Kienzle et al., 2023). In the latter case, the authors assumed an involvement of the alternative starts codons ATT and ATA to expand the peptide (33 instead of the canonical twenty amino acids). To deliver experimental evidence for this assumption, we mined publicly available MS/MS datasets of man (*n* = 48), mouse (*n* = 5) and rat (*n* = 3) using a targeted peptide search with the engine PepQuery. While the MS data of the two rodents completely lacked a confidential SHLP6 fragment, we obtained MS support for the existence of human SHLP6. However, there was no evidence for alternative translation initiation that would result in size expansion. In detail, the same confident peptide fragment “MLDQDIPMVQPLLK” was identified between one to ten times in four of the human data sets (Figure 3). It completely matched to the first 14 amino acids of the mitochondrially encoded human SHLP6 according to UniProt BLAST or Standard Protein BLAST (primary accession A0A3G1DJN1 of UniProtKB and NCBI’s GenBank accession number AMZ80341, respectively), but also to a micropeptide translatable from a NUMT on human chromosome 17 (positions 22,524,419 to 22,524,480 in NC_000017.11).

The PTM datasets contained in PepQuery’s database did not yield any confident fragment for SHLP6.

### 3.3 Steady-state abundance of *mt-Shlp6* transcript during states of hibernation

**FIGURE 4:**
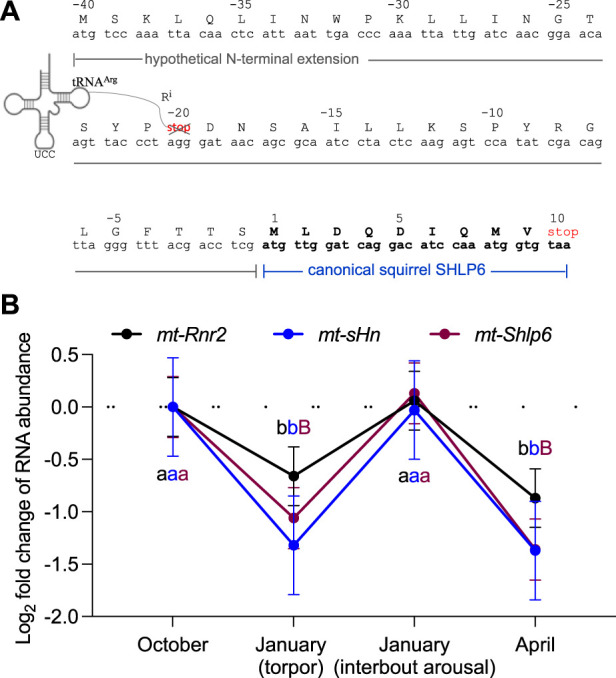
Dynamic abundance alteration of SHLP6-encoding RNA in brown adipose tissue of the thirteen-lined ground squirrel during states of hibernation. **(A)** Deduction of SHLP6 ORF used as “query” sequence in the comparison of steady-state RNA levels. Deduction considered the extended coding repertoire of the mitogenome, here, the import of the cytoplasmic tRNA^Arg^ (AGG) into the mitochondrion (Rubio et al., 2008; Jeandard et al., 2019). As a result, the stop codon that divided the two peptides (in red font), gets converted and the putative SHLP6 peptide expanded. Amino acids are depicted by single-letter code and R^i^ denotes the arginine residue resulting from import of cytoplasmic tRNA^Arg^. **(B)** Temporal pattern of the steady-states of transcripts covering the extended SHLP6-encoding ORF. RNA abundance is expressed as transcripts per million. Error bars depict standard deviations. Wald test implemented by DESeq2 was used to assess significance of differential transcript abundance. Difference among a pair of time points is indicated by a different small or capital letter (small letter: 0.05 > *p* > 0.01, capital letter: *p* < 0.01). Accession number of the public RNA-seq data used for differential analysis of transcript abundance: PRJNA226612.

In general, hibernation-related RNA could be subject to transcriptional and/or post-transcriptional gene regulation (Gillen et al., 2021). Considering the higher incidence of the 9 mer isomer of SHLP6 seen in heterothermic rodents (Section 3.1), we asked whether hibernation would be a phenotype to provide insight into regulation of steady-state RNA level of *mt-Shlp6*. Therefore, we screened a set of public RNA-seq data available for the thirteen-lined ground squirrel. Data covered four tissues, namely, adrenal gland, brain, liver and brown adipose tissue, a thermogenic tissue that uses uncoupled mitochondrial respiration to generate heat instead of ATP, and four hibernation stages, namely, torpor and interbout arousal as well as the time points before and after hibernation. First, we confirmed the reported differential RNA abundance of *mt-sHn* in brown adipose tissue taken from different states of hibernation [(Szereszewski and Storey, 2019), Figure 4]. Second, we considered that the RNA-seq library available for brown adipose tissue contained a read length of ∼100 bp. In order to successfully detect an RNA covering the ORF of *mt-Shlp6*, we questioned whether a longer sequence would exceed the length threshold, thus could serve as appropriate transcript (Figure 4A). For its deduction we considered the extended coding repertoire of the mitogenome. Here, the imported cytoplasmic tRNA^Arg^(AGG) would misread a termination codon that formerly separated two smaller peptides, thus expand the predicted size of the squirrel’s SHLP6. Based on this assumption, we found a significantly reduced level during torpor and in spring compared to the time before entering torpor and the state of interbout arousal (*p* = 1.07e^−5^ and *p* = 0.00036, Figure 4B). Notably, *mt-Shlp6* and its hosting rRNA gene, *mt-Rnr2*, exhibited similar quantitative patterns of transcript alteration. Neither *mt-sHn* nor *mt-Shlp6* transcripts were detectable in the other tissues, likely due to the mismatching (longer) insert size of the RNA-seq libraries used for analysis [∼135 bp (adrenal gland) and ∼250 to 280 bp (brain and liver) instead of ∼100 bp (brown adipose tissue)]. The transcript coding for the short, canonical 9mer of SHLP6 was not detected at all, likely also due to subcritical sequence length for the library. An RNA-seq library composed of shorter sequences that match in length would be a fitting alternative (Chhangawala et al., 2015) to cover the short-sized sORF and sequencing enough of the adapter to be accurately identified and trimmed during data analysis.

### 3.4 Biochemical parameters of SHLP6: secondary structure, hydrophobicity and PTM

**FIGURE 5:**
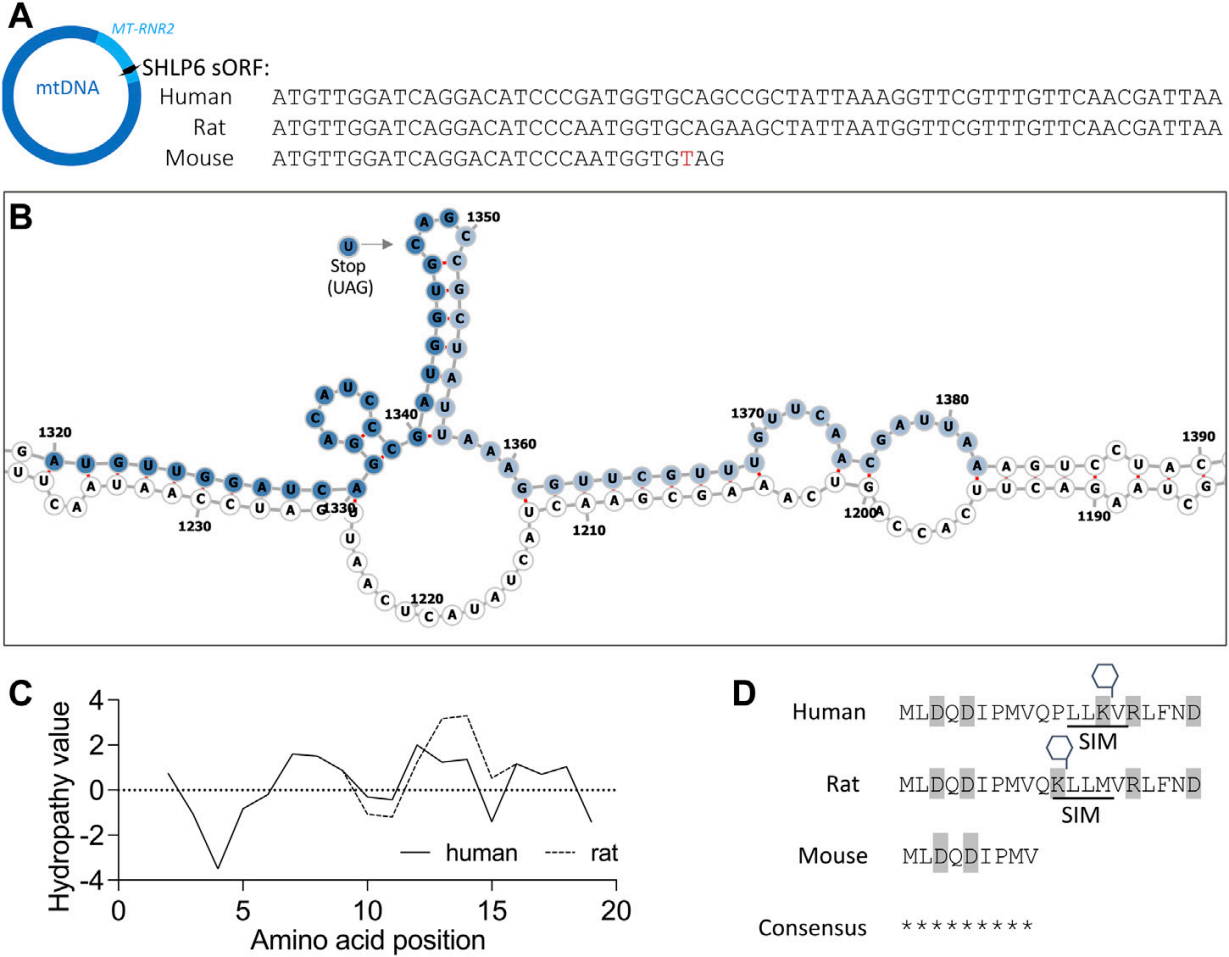
Features of SHLP6 **(A)** The sORF of SHLP6 occurs at two size variants exemplarily represented by human, rat and mouse. Polymorphism m.3,017C>T (red letter) introduces a stop codon in the mouse. In azure: hosting gene *MT-RNR2.* Arrow: sORF orientation. **(B)** Secondary structure predicted by RNAFold3 for the human *MT-RNR2* transcript that hosts the sORF of SHLP6 (nucleotides 1,671 to 3,229 in NC_012920.1). m.3,017C>T depicted by the arrow does not affect folding of the hosting transcript. Dark blue: sORF of the 9mer variant of SHLP6 (30 nucleotides including stop codon); light blue: sORF of the 20mer variant of SHLP6 (63 nucleotides including stop codon). A similar secondary structure was predicted with UNAfold (data not shown). **(C)** Degree of hydrophilicity or hydrophobicity analysed for the amino acids of human and rat SHLP6 [hydropathicity plot in Kyte-Doolittle scale (Kyte and Doolittle, 1982)]. Note that the nine amino acids of mouse SHLP6 are shared by the two 20mer variants of the peptide (see sequence consensus). **(D)** Amino-acid sequences of the SHLP6 length isomers with information on charge (grey background) and putative PTM (SIM: SUMO-interacting motif, hexagon: methylation). Asterisk: identical amino-acid residue. We note that the contribution of the PTMs cannot be predicted in relation to daily or multiday torpor *in silico.*

The mitochondrial SNP target m.3017C>T (28C>T of *mt-Shlp6*) is located within a loop of 16S rRNA according to prediction with the algorithms RNAfold3 and UNAfold, hence would likely not impair the folding of the transcript (Figure 5B). PTM prediction for SHLP6 of human, rat and mouse identified indicated lysine methylation and a SUMO-interacting motif in case of human and rat SHLP6 (Figure 5D).

### 3.5 Prediction of three-dimensional peptide structure for length isoforms of SHLP6

**FIGURE 6:**
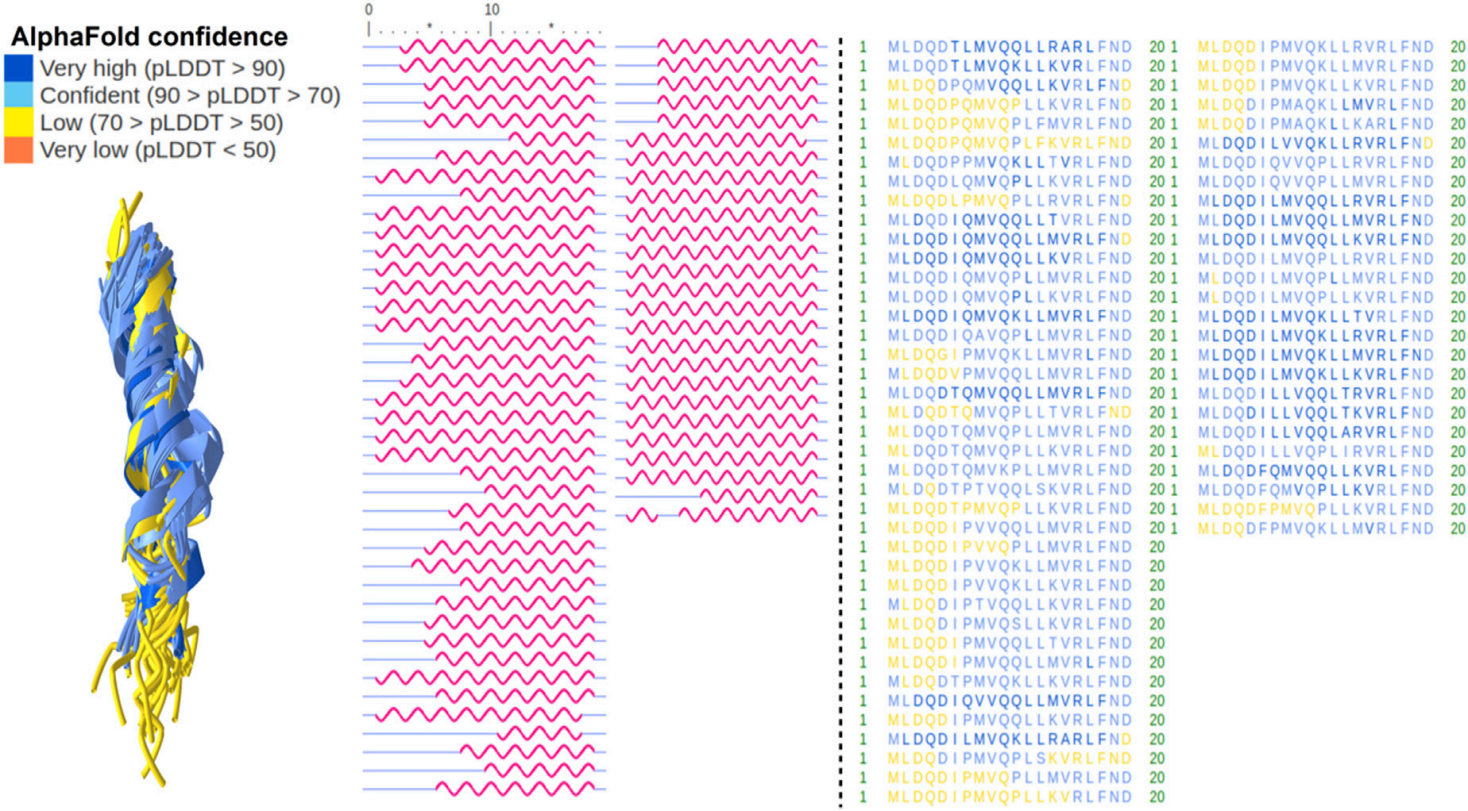
Rodent SHLP6 structures with helices. Three-dimensional peptide structures as predicted by the artificial intelligence system AlphaFold2 are shown on the left with prediction confidence per region indicated by color. The secondary structure for each peptide is shown in the middle with alpha helices indicated in red. Amino acid sequences are shown on the right. All peptides with helices had a length of 20 amino acids. Positions five and 15 of the peptide are highlighted by an asterisk.

**FIGURE 7:**
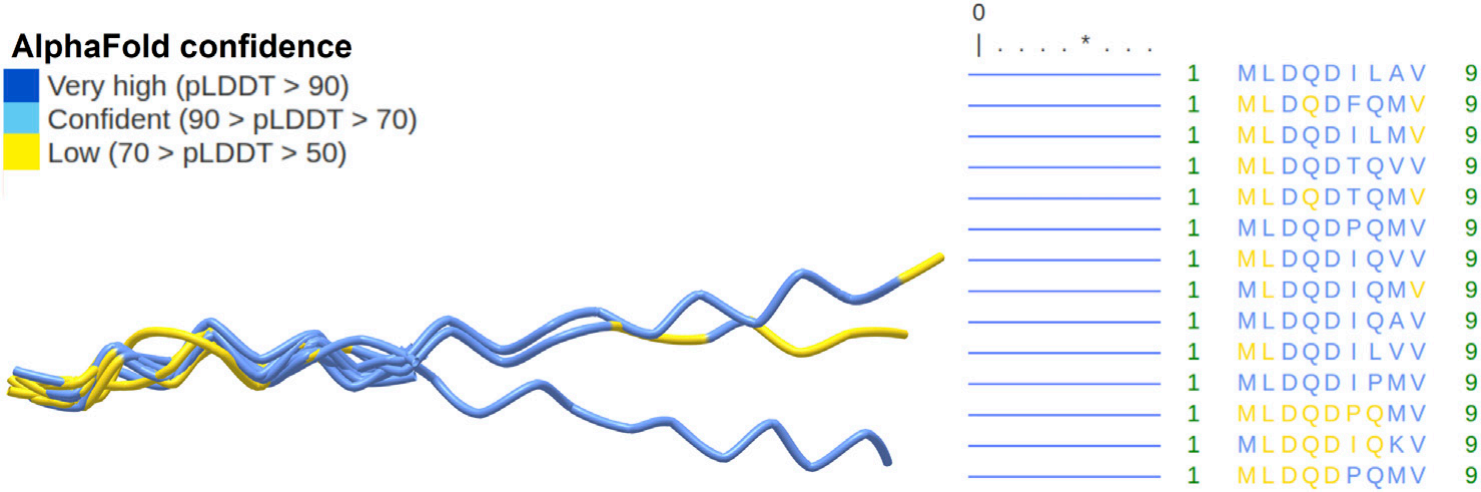
Rodent SHLP6 structures without helices. Three-dimensional peptide structures as predicted by AlphaFold2 are shown on the left with prediction confidence per region indicated by color. No secondary structures were predicted as shown by the horizontal, blue lines. Amino acid sequences are shown on the right. A total of three sequences had a length of 20 amino acids. Only the aligned parts of the peptides are shown. The asterisk highlights position five of the peptide.

Given the relationship of SHLP6 with endothermic thermoregulation (Sections 3.1, 3.2), we tried to add puzzles for its deeper understanding by three-dimensional peptide structure prediction based on the amino acid sequence. It yielded typical foldings for the 9mer and 20mer isoforms of SHLP6 with longer peptides being slightly more heterogeneous (Figures 6, 7). Except for three 20mer isoforms, a single alpha helix that spanned either the entire or almost the entire peptide was predicted. Most structural positions were predicted with high confidence (*pLDDT* score ≥70). None of the structural positions had a very low confidence (*pLDDT* score <50). The lower structural support obtained for the ends of the 20mers (*pLDDT* score <70) was attributed to intrinsic structural disorder (Necci et al., 2021). Secondary-structure components were not found for the 9mer isoforms (Figure 7). The predicted structural variants, helical or non-helical, were clearly associated with the heterothermic rodents (*p* < 0.001, Supplementary Table S4).

It is currently unclear whether reversible PTMs such as lysine methylation, SUMOylation (Figure 2D) or others, alone or in combination rather than SHLP6 size and/or secondary structure are putative components involved in heterothermy regulation. At least for the prediction of folding, we noted a strong dependency on the software version as indicated by the lack of a N-terminal helix in the human SHLP6 structure provided by the AlphaFold Protein Structure Database of EMBL’s European Bioinformatics Institute (Supplementary Figure S2). This can be attributed to differences of the AlphaFold2 algorithms and/or to the ambivalent character of the peptide.

It should be noted that predictions of three-dimensional structure for micropeptides such as SHLP6 must be treated with caution. Micropeptides are in general unstructured and potentially fold upon complex formation [(Kubatova et al., 2020) and communication of Prof. Thomas Martinez]. They resemble intrinsically disordered proteins in their hallmark of marked bias in the amino-acid composition, including a relatively low proportion of hydrophobic and aromatic residues as well as a relatively high proportion of charged and polar residues (Dyson, 2016).

## 4 Discussion

SHLP6 belongs to the class of vertebrate MDPs (Kumagai et al., 2023) and, together with Humanin, it is the second MDP for which the steady-state RNA level was shown to alter in a torpid mammal during hibernation [(Szereszewski and Storey, 2019) and this study].

Here we focussed on the distribution of the *mt-Shlp6* gene polymorphism m.3017C>T across heterothermic and homeothermic species belonging to the taxa Rodentia and Mammalia. It was predicted to cause a length reduction of the MDP from twenty to nine amino acids and to occur at a significantly higher proportion in heterothermic rodents. Mammals in the condition of hibernation or daily torpor showed a significantly higher incidence of the truncating genotype in animals with a lower minimum *T*_*b*_ (*p* = 0.0002). Similarly, non-helical predicted variants of SHLP6 structure were also found to be related to the heterothermic phenotype of the rodents. The concomitant occurrence of length and structural variants hampered clarifying whether alteration of length or structure alone or in combination associates with endothermic thermoregulation. The dichotomy of SHLP6 length was also observed in other orders of the taxon Mammalia (Supplementary Table S5). However, a similar relationship between SHLP6 length and the phenotype of heterothermic thermoregulation was not found (*p* > 0.1). This was attributed to the relatively low number of heterothermic species per order or an alternative adaptive strategy. Summarised, shorter SHLP6 size may mediate tolerance for low minimum *T*_*b*_ and/or better protect during rewarming.

In the future, MDP encoding can more comprehensively be pictured by evaluating the impact of NUMTs (Rubio et al., 2008; Calabrese et al., 2012; Balciuniene and Balciunas, 2019; Lutz-Bonengel et al., 2021; Ng et al., 2022; Wei et al., 2022) and utilization of non-AUG translational start sites for example described for MTALTND4 [(Kienzle et al., 2023); https://www.ncbi.nlm.nih.gov/Taxonomy/Utils/wprintgc.cgi], but not indicated in this study for human SHLP6 based on MS/MS data that would shift the N-terminus of the peptide. Resolution of MS analysis could be enhanced by combining immunoprecipitation with MS, hence help to overcome the failure of MS in detecting mouse and rat SHLP6 (see Sections 3). Another MDP-coding issue represents misreading of termination codons (Beier and Grimm, 2001) facilitated by the import of nucleus-encoded tRNAs (Rubio et al., 2008; Ng et al., 2022). Here, this was assumed for translating SHLP6 of the thirteen-lined ground squirrel and awaits validation along with the proof for transcript sizes of other MDPs, for example, by using Nanopore direct RNA sequencing technology that detects the 5’ end of a transcript at an accuracy of 10–15 nt (Workman et al., 2019). Moreover, the apparent discrepancy between MDPs sizes determined theoretically and experimentally by Western blotting needs clarification. Unresolved cases comprise Humanin (Woodhead et al., 2020), its counterpart in the rat (Caricasole et al., 2002), murine MOTS-c (Cobb et al., 2016; Reynolds et al., 2021) and human MTALTND4 (Kienzle et al., 2023) and Gau for which experimental evidence of MDP size is still lacking (Faure et al., 2011). In case of MTALTND4, for example, neither reduction of disulfide bonds, dephosphorylation nor deglycosylation could remove the molecular weight discrepancy (Kienzle et al., 2023). Mitochondrial protein methylation that might be extensive and comparable to the cellular average (Małecki et al., 2022) and other PTMs should be considered when addressing the striking size discrepancy seen in immunoblots for some MDPs. SUMO that adds a theoretical molecular weight of ∼12 kDa (Daniel et al., 2017), is one of the putative posttranslational modifiers to be tested in this regard. In case of MTALTND4, the issue of the size discrepancy was attributed to the formation of homomultimers (Kienzle et al., 2023). And last but not least, a better understanding of retrograde signalling mediated by the diverse types of RNA that exit the mitochondrion (Tessier et al., 2017; Mathers and Staples, 2019) would be needed. In principle, this could lead to MDP translation according to the nuclear genetic code (Tessier et al., 2017; Mathers and Staples, 2019). The issue of mitochondrial retrograde signalling should further be extended to non-coding RNA regulators of mitochondrial origin such as ASncmtRNA-1 and ASncmtRNA-2 that together with *MT-SHLP6* map to the same rRNA gene. Theses loop-containing, polyadenylated antisense transcripts are widely abundant in proliferating cells, ubiquitously localised in the nucleus and modulate nuclear gene expression in a retrograde manner (Burzio et al., 2009; Ren et al., 2023). Considering that *mt-Shlp6* was found to be dynamically regulated in brown adipose tissue of the thirteen-lined ground squirrel across hibernation states (Figure 4), the existence of ASncmtRNA-1 and ASncmtRNA-2 in hibernating model species and their abundance changes during daily torpor or the stages of hibernation are of special interest (Burzio et al., 2009). They should also be analysed with regard to the finding that most long non-coding RNAs contain sORFs, hence, the potential to encode functional micropeptides (Pan et al., 2022).

It should also be addressed whether SHLP6 upregulation before torpor and during interbout arousal supports clearance of metabolites that accumulated due to torpor-induced low blood flow considering retrograde signalling by one or several MDPs as well as *cis*-acting regulatory elements and additional mtDNA polymorphisms that are related to heterothermy given the importance of the mitochondrial oxidative phosphorylation system in energy and heat production. Insight into the role of SHLP6 in endothermic thermoregulation can further be substantiated by increasing the number of SHLP6-coding sORFs, the amount of behavioural data with respect to heterothermic or homeothermic thermoregulation of rodents, and by deciphering the detailed mode(s) of action.

## 5 Conclusion and future perspectives

This study highlighted the conservation and variation of the apoptotic regulator peptide SHLP6 across Rodentia and established the connection between heterothermy and a member of the class of MDPs. It also provided the gold-standard level of proof based on MS for the canonical variant of human SHLP6, and added evidence to the involvement of the peptide in hibernation physiology by demonstrating differential transcript abundance for a hibernating model rodent. SHLP6, putatively an extracellular communicator (Woodhead et al., 2020), has likely a multi-facetted impact. This can be assumed based on its apoptotic function and the variation in micropeptide size, with one isoform being more favourable at a lower minimum *T*_*b*_ of a heterothermic mammal. Reduction of SHLP6 size could modify the potential for oligomerisation (Kienzle et al., 2023), extracellular and/or intracellular signalling (Hartford and Lal, 2020), and/or the number of binding sites for putative PTM switches that could act as a checkpoint to guarantee physiological safeness (Lee et al., 2015; Dai et al., 2022). Further analysis of SHLP6 size variation might focus on the stress perceived during cold exposure. Perceived stress is associated with altered metabolic activity of the amygdala, a brain region involved in stress. It can be measured using ^18^F-fluorodeoxyglucose positron emission tomography/computed tomography (Tawakol et al., 2017). For example, genetically closely related rodents (e.g., of the genus *Rattus*) that differ in the length of their SHLP6 isomers could be compared. In addition, species such as the gray squirrel (*Sciurus carolinensis*) having size variants encoded by mitochondrial and nuclear DNA, are promising models. *In vivo* experimentation could focus on the metabolic outcome of the 9mer of SHLP6 administered exogenously (Merry et al., 2020; Kim et al., 2023).

A better understanding of SHLP size isomerisation and regulation can be of importance for therapeutic hypothermia, also known as targeted temperature management (Bernard et al., 2002; Garcia-Rubira et al., 2023). It is intentionally used in certain clinical situations to slow the metabolism and help reduce the risk of tissue damage following periods of insufficient blood flow, most commonly after resuscitation from cardiac arrest (Arrich et al., 2023). While therapeutic hypothermia has potential benefits, it also carries risks such as infection, coagulopathy, arrhythmias, and electrolyte imbalances (Geurts et al., 2014; Karnatovskaia et al., 2014; Wang et al., 2015). Therefore, its use must be carefully managed and a protective drug therapy might be of value.

## Data Availability

The datasets presented in this study can be found in online repositories. The names of the repository/repositories and accession number(s) can be found in the article/Supplementary Material.
