# Supplementary material for: Mitochondrial polymorphism m.3017C>T of SHLP6 relates to heterothermy
Source: Front Physiol. 2023 Aug 21;14:1207620. doi: 10.3389/fphys.2023.1207620 (PMC10478271; doi:10.3389/fphys.2023.1207620)
Supplement: Supplementary file 2 [file Table1.pdf]

Table S1 | Putative roles of MDPs in heterothermy

MDP	Putative function in heterothermic thermoregulation	Evidence
Gau	Unknown	No
Humanin	Reversion of neurological degeneration	(Szereszewski and Storey 2019)
	Cell protection during torpor	No
MOTS-c	Thermogenic gene upregulation in BAT	(Lu et al. 2019)
	Cell protection during torpor	No
	Lipid metabolism, insulin resistance	No
MTALTND4	Response to low oxygen availability during torpor	No
SHLP1	Unknown	No
SHLP2	Thermogenesis in interscapular BAT	(Kim et al. 2023)
SHLP2 and SHLP3	Cell protection during torpor	No
	Lipid metabolism, insulin resistance	No
	Clearance of accumulated ROS	No
	Facilitation of arousals	No
SHLP3	Cell protection during torpor	No
	Clearing of accumulated ROS	No
SHLP4	Restoration during arousals/after torpor	No
SHLP5	Unknown	No
SHLP6	Organ shrinkage before torpor	No
	Thermoregulation	No
SHMOOSE	Cell protection during torpor	No

REFERENCES

Kim SK, Tran LT, NamKoong C, Choi HJ, Chun HJ, Lee YH, Cheon M, Chung C, Hwang J, Lim HH, et al. 2023. Mitochondria-derived peptide SHLP2 regulates energy homeostasis through the activation of hypothalamic neurons. Nat Commun. Jul 19;14:4321. Epub 20230719.

Lu H, Tang S, Xue C, Liu Y, Wang J, Zhang W, Luo W, Chen J. 2019. Mitochondrial-Derived Peptide MOTS-c Increases Adipose Thermogenic Activation to Promote Cold Adaptation. International journal of molecular sciences.20:2456-2456.

Szereszewski KE, Storey KB. 2019. Identification of a prosurvival neuroprotective mitochondrial peptide in a mammalian hibernator. Cell Biochemistry and Function.37:494-503.

Varadi M, Anyango S, Deshpande M, Nair S, Natassia C, Yordanova G, Yuan D, Stroe O, Wood G, Laydon A, et al. 2022. AlphaFold Protein Structure Database: massively expanding the structural coverage of protein-sequence space with high-accuracy models. Nucleic Acids Res. Jan 7;50:D439-D444.
