# Supplementary material for: Mitochondrial polymorphism m.3017C>T of SHLP6 relates to heterothermy
Source: Front Physiol. 2023 Aug 21;14:1207620. doi: 10.3389/fphys.2023.1207620 (PMC10478271; doi:10.3389/fphys.2023.1207620)
Supplement: Supplementary file 3 [file DataSheet1.PDF]

SUPPLEMENTARY FIGURES

Mitochondrial polymorphism m.3017C>T of SHLP6 relates to heterothermy

Sarah V. Emser*, Clemens P. Spielvogel, Eva Millesi, Ralf Steinborn

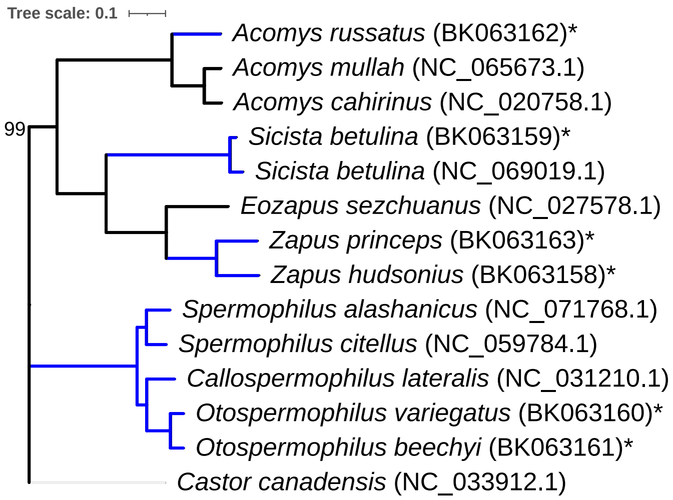

Figure S1 | Phylogenetic reconstruction of novel mitogenomes using IQ-Tree maximum likelihood analysis and mitogenomes without partitioning. Tree root: *Castor canadensis*; bootstrap replicates: 1,000; bootstrap support values for branching: displayed if < 100. In brackets: accession numbers of NCBI's GenBank. Mitogenomes extracted in this study are highlighted by an asterisk. Species included in the tree are heterothermic (blue line) except of the outgroup and the three species *Acomys mullah*, *A. cahirinus* and *Eozapus sezchuanus* for which the mode of endothermic thermoregulation is not explored (black line).

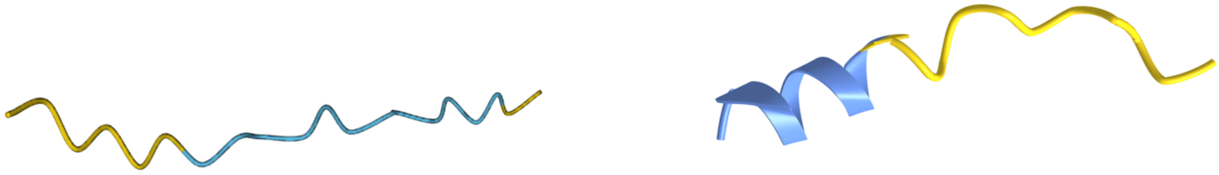

Figure S2. Three-dimensional structure predictions for human SHLP6 based on the artificial intelligence system AlphaFold2. **Left:** "Spaghetti"-like structure provided by the AlphaFold DB version 2022-11-01 at EMBL's European Bioinformatics Institute (EMBL-EBI) that was created with the AlphaFold Monomer v2.0 pipeline (Varadi et al. 2022) (https://alphafold.ebi.ac.uk/entry/A0A3G1DJN1). **Right:** N-terminal helix predicted in this study. UniProt accession number of human SHLP6: A0A3G1DJN1.

REFERENCE

Kim SK, Tran LT, NamKoong C, Choi HJ, Chun HJ, Lee YH, Cheon M, Chung C, Hwang J, Lim HH, et al. 2023. Mitochondria-derived peptide SHLP2 regulates energy homeostasis through the activation of hypothalamic neurons. Nat Commun. Jul 19;14:4321. Epub 20230719.

Lu H, Tang S, Xue C, Liu Y, Wang J, Zhang W, Luo W, Chen J. 2019. Mitochondrial-Derived Peptide MOTS-c Increases Adipose Thermogenic Activation to Promote Cold Adaptation. International journal of molecular sciences.20:2456-2456.

Szereszewski KE, Storey KB. 2019. Identification of a prosurvival neuroprotective mitochondrial peptide in a mammalian hibernator. Cell Biochemistry and Function.37:494-503.

Varadi M, Anyango S, Deshpande M, Nair S, Natassia C, Yordanova G, Yuan D, Stroe O, Wood G, Laydon A, et al. 2022. AlphaFold Protein Structure Database: massively expanding the structural coverage of protein-sequence space with high-accuracy models. Nucleic Acids Res. Jan 7;50:D439-D444.
